# Culturally focused video education targeted toward Hispanic patients increases melanoma knowledge and sun-protective behaviors after 12 months

**DOI:** 10.1016/j.jdin.2025.09.011

**Published:** 2025-10-09

**Authors:** Andy Ho, Sarah Amjad, Adina Greene, Richard J. Butterfield, Nan Zhang, Aaron R. Mangold, Mary-Ellen Brown, Collin M. Costello

**Affiliations:** a Mayo Clinic Alix School of Medicine, Phoenix, Arizona; b Department of Dermatology, Mayo Clinic, Scottsdale, Arizona; c University of Arizona College of Medicine-Phoenix, Phoenix, Arizona; d Department of Quantitative Health Sciences, Mayo Clinic, Scottsdale, Arizona; e The University of Texas at Austin Steve Hicks School of Social Work, Austin, Texas

**Keywords:** health disparate, melanoma, minority and vulnerable populations, minority groups, minority health

*To the Editor:* Melanoma is more commonly diagnosed in non-Hispanic Whites than in Hispanics.^1^ However, Hispanics, regardless of Fitzpatrick type, have a risk for acral lentiginous melanoma.^2^ Culturally focused education for the Hispanic population has been shown to improve knowledge of melanoma, sun-protective behaviors, and friend, family, or self-skin examinations.^3^ We have previously evaluated knowledge retention of a bilingual, culturally focused melanoma educational video versus text-based educational material, with a follow-up duration limited to 3 months, with another similar study also limited to a 3-month follow-up.^4^^,^^5^ The goal of this study is to evaluate outcomes of a video-based Hispanic melanoma educational program at 12 months.

The Mayo Clinic Institutional Review Board approved this study. A search of Mayo Data Explorer identified 89,778 patients who self-identified as Hispanic or Latino who were then emailed a link to a bilingual video-based melanoma education including information on melanoma risk factors for the Hispanic population, self-skin examinations, and sun-protective behaviors embedded within a Qualtrics survey with the option to opt in to receive a text reminder to rewatch the video every 3 months. Knowledge was evaluated using a Melanoma Knowledge Score (MKS). See Supplementary Methods, available via Mendeley at https://data.mendeley.com/datasets/kkfwkzyxnz/1.

1269 patients completed the survey, 810 (63.8%) completed the 3-month follow-up survey, and 653 (47.0%) completed the 12-month follow-up survey. Mean MKS pre-education was 9.6 compared to 12.3 posteducation (*P* < .0001), 11.3 after 3 months (*P* < .0001), and 11.3 after 12 months (*P* < .0001) (Table I; Fig 1). Of the 1142 (90.1%) patients who opted in to receive the text reminder, 711 (62.3%) completed the 3-month follow-up survey, and 408 (57.3%) of those patients viewed the online website at 3-month follow-up. At 12 months, 571 (50%) of the patients who opted in for text reminders completed the follow-up survey, with 355 (62.2%) of those patients viewing the website (Table I). Rewatching the video did not improve MKS; however, those who rewatched had improved confidence in checking for signs of skin cancer at 3 months (*P* < .001) and 12 months (*P* = .025), and in encouraging friends and family to get a skin examination at 3 months (*P* = .03) and 12 months (*P* = .006).

**Table I:** Melanoma Knowledge Score and text message reminders

	Pre-educational video	Posteducational video	3-month follow-up	12-month follow-up
	(*N* = 1269)	(*N* = 1269)	(*N* = 810)	(*N* = 653)
Melanoma Knowledge Score				
*N* (missing)	1269 (0)	1269 (0)	810 (0)	653 (0)
Mean (SD)	9.6 (2.9)	12.3 (2.6)	11.3 (2.6)	11.3 (2.6)
Median (IQR)	10 (8, 12)	13 (11, 14)	12 (10, 13)	12 (10, 13)
Range	−2.0, 15.0	0.0, 15.0	0.0, 15.0	0.0, 15.0
Melanoma Knowledge Score by website viewing, mean (*P* value)	N/A	N/A	No = 11.1, Yes = 11.4 (*P* = .218)	No = 11.4, Yes = 11.3 (*P* = .793)
Would it be okay to text you a reminder to perform a self-skin examination every 3 months?, *n* (%)				
Missing	1	N/A	N/A	N/A
Yes	1142 (90.1%)	N/A	N/A	N/A
No	126 (9.9%)	N/A	N/A	N/A
After receiving the reminder text message, did you perform a self-skin examination?, *n* (%)				
Missing	N/A	N/A	99 (12.2%)	87 (13.3%)
Yes, I performed a self-skin examination after every text message reminder.	N/A	N/A	284 (35.1%)	249 (38.1%)
Yes, I performed a self-skin examination after some of the text message reminders.	N/A	N/A	177 (21.9%)	201 (30.8%)
No, I did not.	N/A	N/A	250 (30.8%)	116 (17.8%)
Did you view the online website from the link that was texted to you?, *n* (%)				
Missing	N/A	N/A	98 (12.1%)	82 (12.6%)
Yes, I viewed the website and performed a self-skin examination.	N/A	N/A	309 (38.1%)	290 (44.4%)
Yes, I viewed the website but did not perform a self-skin examination.	N/A	N/A	99 (12.2%)	65 (9.95%)
No	N/A	N/A	304 (37.5%)	216 (33.1%)

*IQR*, Interquartile range; *N/A*, not applicable; *SD*, standard deviation.

**Fig 1:**
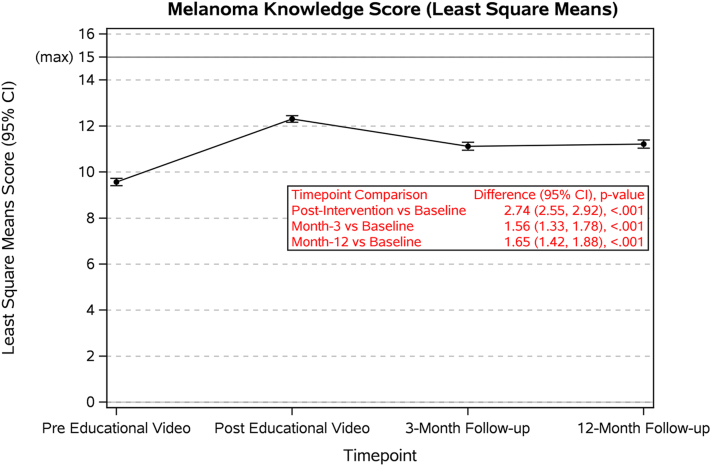
Melanoma Knowledge Score at baseline, posteducational video, 3-month follow-up, and 12-month follow-up.

MKS peaked immediately after the education but remained consistently elevated at 3 and 12 months compared to baseline, indicating that this educational method provides durable knowledge retention. Participants with higher MKS were less likely to use tanning beds and were more likely to wear sunscreen and sun protective clothing outside.^3^ There was no significant difference in MKS between patients who reviewed the online website and those who did not at both 3 months and 12 months, suggesting that repeat education was not needed to maintain knowledge gain, but did improve confidence and behavior. Limitations include a small number of survey respondents and a limited number of those completing the 12-month follow-up, potentially limiting generalizability as those engaged to complete a long-term survey could be more engaged to interact and learn from the educational materials. Our study demonstrated that culturally focused melanoma educational videos targeting Hispanic patients can enhance melanoma knowledge immediately after education and at 3 and 12 months posteducation.

## Conflicts of interest

Dr Costello has no relevant disclosures. Unrelated to this study, he is an investigator for Merck and was an investigator for DeepX Health within the last 12 months. Dr Mangold has no relevant disclosures. Unrelated to this study, he has consulted for Phlecs BV, Kyowa, Eli Lilly, Momenta, UCB, and Regeneron in the past, more than 24 months ago. He has consulted for Incyte, Soligenix, Clarivate, Argenyx, and Bristol Myers Squibb in the past, less than 12 months ago. He consults for PPD, Nuvig, Tourmaline Bio, Janssen, and Boehringer Ingelheim currently. He consults for Regeneron and Pfizer currently with payments to the institution. He has grant support from Kyowa, Miragen, Regeneron, Corbus, Pfizer, Incyte, Eli Lilly, Argenx, Palvella, AbbVie, Priovant, Bristol Myers Squibb, and Merck in the last 24 months. Beyond 24 months, grant support has come from Sun Pharma, Elorac, Novartis, and Janssen. He has received royalties from Adelphi Values and Clarivate. His current patents include Methods and Materials for assessing and treating cutaneous squamous cell carcinoma (PCT/US2023/078902); Use of Oral Jaki in Lichen Planus PCT/US2024/020149; and Topical Ruxolitinib in Lichen Planus PCT/US2021/053149, 2023-520085, and 21805700.8, respectively. Methods and Materials for Treating Lichen Planopilaris (Registration Number: 53103), Machine-Learning Models for Tumor Grading Using Rank-Aware Contextual Reasoning on Whole Slide Images (63/616287). Andy Ho, Sarah Amjad, Dr Greene, Richard J. Butterfield III, Nan Zhang, and Dr Brown have no conflicts of interest to declare.
